# Phase I-II study using DeltaRex-G, a tumor-targeted retrovector encoding a cyclin G1 inhibitor for metastatic carcinoma of breast

**DOI:** 10.3389/fmmed.2023.1105680

**Published:** 2023-05-18

**Authors:** Howard W. Bruckner, Sant P. Chawla, Nadezhda Omelchenko, Don A. Brigham, Erlinda M. Gordon

**Affiliations:** ^1^ Bruckner Oncology, New York, NY, United States; ^2^ Cancer Center of Southern California, Santa Monica, CA, United States; ^3^ Aveni Foundation, Santa Monica, CA, United States

**Keywords:** DeltaRex-G, cancer gene therapy, cell cycle control, CCNG1, cyclin G1 inhibitor, metastatic breast cancer, DeltaVax, immunotherapy

## Abstract

**Background:** Metastatic breast cancer is associated with a poor prognosis and therefore, innovative therapies are urgently needed. Here, we report on the results of a Phase I-II study using DeltaRex-G for chemotherapy resistant metastatic carcinoma of breast.

**Patients and Methods:** *Endpoints:* Dose limiting toxicity; Antitumor activity. *Eligibility*: ≥18 years of age, pathologic diagnosis of breast carcinoma, adequate hematologic and organ function. *Treatment*: Dose escalation of DeltaRex-G 1-4 x 10^11^cfu intravenously thrice weekly x 4 weeks with 2-week rest period. Treatment cycles repeated if there is ≤ Grade 1 toxicity until disease progression or unacceptable toxicity. *Safety:* NCI CTCAE v3 for adverse events reporting, vector related testing. *Efficacy:* RECIST v1.0, International PET criteria and Choi criteria for response, progression free and overall survival.

**Results:** Twenty patients received escalating doses of DeltaRex-G from 1 × 10^11^ cfu to 4 × 10^11^ cfu thrice weekly for 4 weeks with a 2-week rest period. *Safety*: ≥ Grade 3 treatment-related adverse event: pruritic rash (*n* = 1), no dose limiting toxicity, no replication-competent retrovirus, nor vector-neutralizing antibodies detected. No vector DNA integration was observed in peripheral blood lymphocytes evaluated. *Efficacy*: by RECIST v1.0: 13 stable disease, 4 progressive disease; tumor control rate 76%; by PET and Choi Criteria: 3 partial responses, 11 stable disease, 3 progressive disease; tumor control rate 82%. Combined median progression free survival by RECIST v1.0, 3.0 months; combined median overall survival, 20 months; 1-year overall survival rate 83% for Dose Level IV. Biopsy of residual tumor in a participant showed abundant CD8^+^ killer T-cells and CD45^+^ macrophages suggesting an innate immune response. Two patients with pure bone metastases had >12-month progression free survival and overall survival and are alive 12 years from the start of DeltaRex-G therapy. These patients further received DeltaRex-G + DeltaVax for 6 months.

**Conclusion:** Taken together, these data indicate that 1) DeltaRex-G has a distinctively high level of safety and exhibits anti-cancer activity, 2) PET/Choi provide a higher level of sensitivity in detecting early signs of tumor response to DeltaRex-G, 3) DeltaRex-G induced 12- year survival in 2 patients with pure bone metastases who subsequently received DeltaVax immunotherapy, and 4) DeltaRex-G may prove to be a biochemical and/or immune modulator when combined with other cancer therapy/immunotherapy.

## 1 Introduction

According to the American Cancer Society statistics 2019, 271,270 persons are diagnosed with breast cancer and 42,260 persons die of the disease every year (Siegel et al., 2019). In recent years, precision medicine has come of age and the treatment of metastatic breast cancer now includes hormone therapy, targeted therapy, and immunotherapy with or without chemotherapy. Chemotherapy drugs include taxanes such as paclitaxel, docetaxel, albumin-bound paclitaxel, ixabepilone, and eribulin; anthracyclines such as doxorubicin, liposomal doxorubicin, and epirubicin, and platinum agents such as cisplatin and carboplatin; vinorelbine, capecitabine and gemcitabine (Jagsi et al., 2019; NCCN, 2021).

For hormone therapy, selective estrogen receptor modulators such as tamoxifen and selective estrogen receptor degrader such as fulvestrant have been combined with a selection of CDK4/6 inhibitors in palliative combination with hormone therapy (HT) initially and with second line CDK4/6 inhibitors and HT in sequence or a P13K inhibitor to treat metastatic breast cancer after other hormone treatments have failed (Mukohara, 2015; NCCN, 2021). Palbociclib, ribociclib, and abemaciclib block CDK4 and CDK6 cyclin dependent kinases (CDKs). These drugs are FDA approved for women with advanced hormone receptor-positive, HER2-negative breast cancer, usually together with an aromatase inhibitor or fulvestrant for women who have gone through menopause. Abemaciclib can also be used in women who have previously been treated with hormone therapy and chemotherapy (Ma and Sparano, 2021; NCCN, 2021). Recent evidence supports a positive role of hormone therapy in ER + HER2+ metastatic breast cancer (Cardoso, 2020).

About 30%–40% of breast cancers have a mutated PIK3CA gene. Alpelisib inhibits the PI3K protein found in cancer cells and is utilized in combination with fulvestrant for the management of advanced hormone receptor-positive, HER2-negative breast cancer in postmenopausal women. The treatment is specifically for those with a PIK3CA gene mutation, whose cancer has progressed while on or after receiving an aromatase inhibitor (Mukohara, 2015; NCCN, 2021). There are evolving safety guidelines on the use of drugs that target PIK3CA (NCCN, 2021). For HER2 positive breast cancer, a number of drugs have been developed that inhibit the HER2 protein kinase (Moasser and Krop, 2015; NCCN, 2021). Lapatinib, a protein kinase inhibitor is used to treat advanced breast cancer, usually with trastuzumab and capecitabine. Tucatinib kinase inhibitor is used to treat advanced breast cancer after one other anti-HER2 drug has failed usually with trastuzumab and capecitabine. Neratinib is another kinase inhibitor that is given with capecitabine for metastatic disease after 2 other anti-HER2 targeted drugs have been tried (Chan et al., 2016; NCCN, 2021).

Everolimus blocks the mammalian target of rapamycin (mTOR) pathway and has antiangiogenic properties. This drug is approved for menopausal women who have advanced hormone receptor-positive, HER2-negative breast cancer. It is used with the aromatase inhibitor, exemestane, for women whose cancers have grown while on either letrozole or anastrozole. It might also be used with fulvestrant (Baselga et al., 2012; NCCN, 2021). Olaparib and talazoparib are PARP inhibitors. PARP and BRCA proteins help repair damaged DNA (Henry et al., 2020; NCCN, 2021). However, *BRCA* (*BRCA1* and *BRCA2*) gene mutations impair the DNA repair process. PARP inhibitors work by blocking the mutated PARP proteins. Olaparib and talazoparib are approved for the treatment of advanced or metastatic, HER2-negative breast cancer in women who carry a BRCA mutation, who have failed chemotherapy. If the tumor is hormone receptor-positive, olaparib can also be used in women who have failed hormone therapy.

For advanced triple-negative breast cancer (TNBC), sacituzumab govitecan, an antibody-drug conjugate (ADC), i.e., a monoclonal antibody joined to a chemotherapy drug can be used as monotherapy for advanced TNBC that failed at least 2 other chemo regimens (Bardia et al., 2017; NCCN, 2021). Pembrolizumab is a type of monoclonal antibody that hinders the programmed death-1 (PD-1) immune checkpoint. These drugs, by obstructing PD-1, enhance the immune response against breast cancer cells and can potentially impede the growth or reduce the size of tumors. In some cases, it is utilized in conjunction with chemotherapy to combat triple-negative breast cancer and is indicated for advanced or recurrent TNBC with evolving data for a possible role as neoadjuvant therapy (Schmidt et al., 2018).

Even though therapies such as chemotherapy, endocrine therapy, and targeted therapy have notably enhanced outcomes for women with early-stage breast cancer, the survival rate for women with metastatic disease (mBC) is still unsatisfactory, with a mere 29% survival rate over 5 years. This highlights the urgency to develop innovative therapeutic approaches (ACS, 2022). Here, we report on the results of a Phase I/II study using DeltaRex-G, a tumor-targeted retrovector bearing a CCNG1 inhibitor gene for chemotherapy resistant advanced carcinoma of breast.

## 2 Patients and methods

### 2.1 Study objectives

The primary goal is to identify the toxicity that limits dosage (DLT) and the highest dose that can be administered without causing treatment related serious adverse reactions (MTD) of DeltaRex-G administered as intravenous infusions. The secondary objectives are 1) to evaluate the potential of DeltaRex-G for evoking vector antibodies, recombination events, and unwanted vector integration in nontarget organs, and 2) to identify antitumor activity of DeltaRex-G.

### 2.2 Study drug and United States Food and Drug Administration (FDA)-approved vector production

**FIGURE 1:**
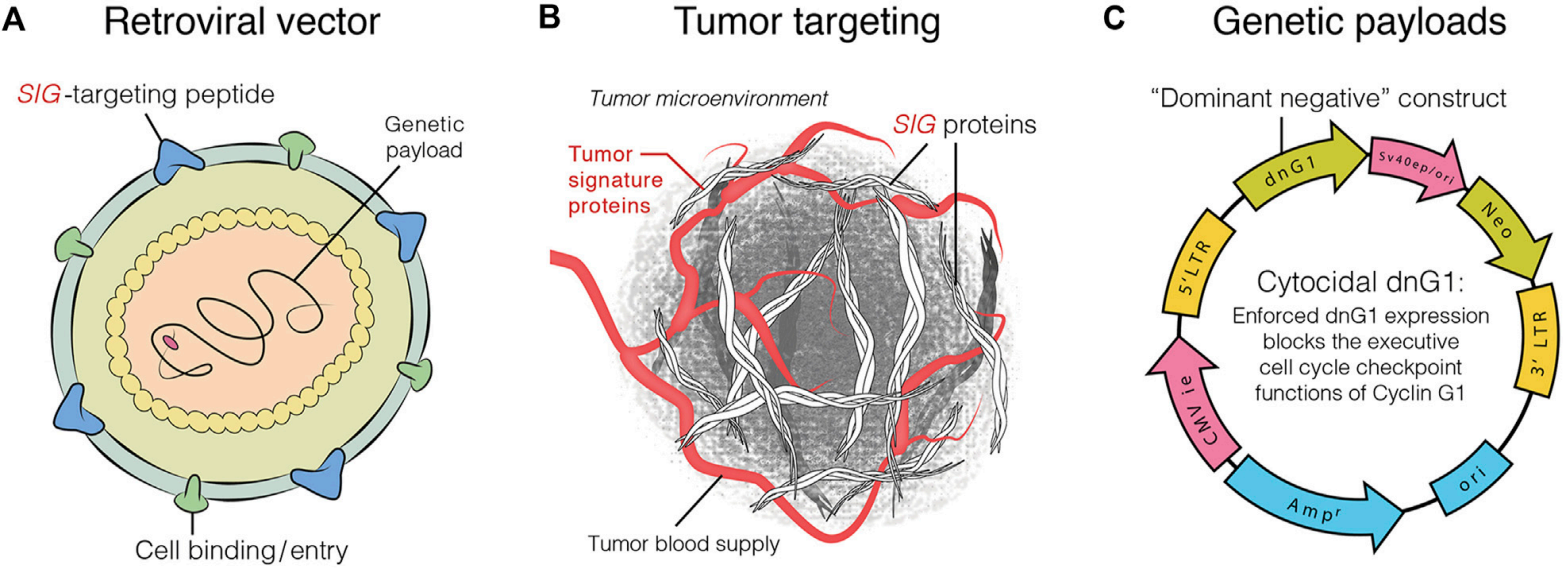
Graphic illustration of DeltaRex-G vector. The DeltaRex-G vector displaying a *SIG* targeting peptide **(A)**, for binding to Signature (Sig) Proteins in the tumor microenvironment [TME] **(B)**, and encoding a dominant negative human cyclin G1 inhibitor gene **(C)**. Injected intravenously, DeltaRex-G nanoparticles seek out and bind to abnormal *SIG*proteins in the TME which augments effective vector concentration in tumors (Chawla et al., 2019).

DeltaRex-G is an amphotropic retrovector based on murine leukemia virus (MLV) that does not replicate and is designed to target abnormal Signature (SIG) collagenous proteins in the tumor microenvironment. It achieves this by displaying a collagen-binding motif referred to as cryptic Signature (SIG) on its gp70 surface membrane (TME; Hall et al., 2010; Stendahl Dy et al., 2018), and encoding a dominant negative mutant construct of human cyclin G1 (Gordon et al., 2018; Xu et al., 2001). The vector comprises a neomycin resistance (neo^r^) gene that is controlled by the SV40 early promoter (Chawla et al., 2019; Figure 1). DeltaRex-G is produced by transiently co-transfecting human embryonic kidney 293 T-cells with 3 proprietary plasmid DNAs. Details of the production and evaluation of the clinical vector are available in other sources (Galanis et al., 2008; Chawla et al., 2009).

The final product exhibits a vector titer of 5 × 10^9^ colony forming units (cfu) per milliliter, a biologic potency of 50%–70% growth inhibitory activity in target cancer cells, less than 550 bp residual DNA, no detectable E1A or SV40 large T antigen, and no detectable replication competent retrovirus (RCR), in compliance with FDA recommendations for retroviral vector-based gene therapy products. The vector formulation is stored in aliquots of 23 mL in a 30 mL glass vial and kept frozen at −70^o^ to −90°C until used. The DeltaRex-G vector is prepared for patient administration by quickly thawing it in a 34°–37°C water bath, either in the vial or cryobag. The vector is thawed 15–30 min before being given to the patient via intravenous infusion at a rate of 4 mL per minute. During handling and disposal of the vector, all personnel comply with Biosafety Level 2 regulations in accordance with the National Institutes of Health Guidelines for Research Involving Recombinant DNA molecules.

### 2.3 Vector-related testing and biodistribution studies

The evaluation process involved conducting several tests, including detecting the presence of anti-vector antibodies in the patient’s serum, testing for the presence of replication-competent retroviruses (RCRs), and performing vector DNA integration studies in the patient’s peripheral blood lymphocytes. The methods used for these tests were described previously (Galanis et al., 2008).

### 2.4 Study design

Using the Cohort of Three design (Storer, 1989), three patients are treated at each dose level with expansion to 6 patients per cohort if dose limiting toxicity (DLT) is observed in any 1 of the first 3 patients at each dose level. The maximum tolerated dose (MTD) was the dose level wherein none of the three patients or at most one out of six patients experienced a DLT and wherein at least two patients experienced a DLT at the subsequent higher dose level. A DLT was defined as any National Cancer Institute Common Toxicity Criteria for Adverse Events (NCI CTCAE, 2006) Grade 3, 4, or 5 adverse event (AE) that could be related to the study drug is categorized as possibly, probably, or definitely, with the exception of Grade 3 absolute neutrophil count that lasts less than 72 h; Grade 3 alopecia; or any Grade 3 or higher incidence of nausea, vomiting, or diarrhea in a patient who did not receive maximal supportive care (NCI CTCAE v3).

Adaptive design: The Phase II part of the study was that part in which patients who had no or resolved Grade 1 toxicity were eligible to receive extra cycles of therapy. Protocol Amendments I and II permitted an intra-patient dose escalation up to Dose Level II for patients who did not experience any toxicity or whose toxicity had reduced to Grade 1 or lower, if safety had been confirmed for a higher dose level in a Phase I/II study of sarcoma conducted concurrently (Chawla et al., 2009)^.^ Furthermore, it was possible to expand each cohort in the study to 6 or 7 patients if significant biologic activity (stable disease or better) was noted at each dose level. The principal investigator had the authority to suggest surgical resection or debulking after a minimum of one cycle of treatment. The initial evaluation of the response was performed using the Response Evaluation Criteria in Solid Tumors (RECIST) (Therasse et al., 2000). Furthermore, the International Positron Emission Tomography (PET) criteria (Young et al., 1999) and a modified RECIST as described by Choi et al. (2007) were used as additional evaluations. Safety and efficacy analyses were conducted by the site Principal Investigators (HWB and SPC).

#### 2.4.1 Inclusion criteria

Patients included in the study had to have a confirmed pathologic diagnosis of advanced or metastatic chemotherapy resistant cancer of breast that was determined through histological or cytological examination, be ≥ 18 years of age, have an Eastern Cooperative Oncology Group (ECOG) performance score of 0–1, and acceptable hematologic, hepatic, and kidney function.

#### 2.4.2 Exclusion criteria

Patients with human immunodeficiency virus, hepatitis B virus, or hepatitis C virus positivity, medical or psychiatric conditions that could compromise proper adherence to the protocol, and patients who refuse to use effective contraception during the treatment with DeltaRex-G and for 6 weeks following treatment completion were excluded.

### 2.5 Statistical analysis

Safety evaluable population: Intention-to-Treat (ITT) population were all patients who received at least one dose of DeltaRex-G: 20 patients (used for safety and overall survival). Efficacy Evaluable Population: Modified Intention-to-Treat (mITT) population were all patients who received at least one treatment cycle and had a follow-up PET-CT scan. Seventeen patients in the mITT group were used for response, progression-free survival (PFS) and overall survival (OS).

#### 2.5.1 Safety analysis

Pretreatment evaluation included history, physical exam, and laboratory tests. These tests included a complete blood count with differential and platelet count, and a serum chemistry panel that measured levels of aspartate transaminase, alanine transaminase, alkaline phosphatase, creatinine, and total bilirubin. The evaluation also involved an assessment of the patient’s coagulation status by testing prothrombin time, international normalized ratio, and activated partial thromboplastin time. Furthermore, patients were tested for the presence of human immunodeficiency virus (HIV), hepatitis B virus, and hepatitis C virus. All patients had a complete blood count and serum chemistry panel performed weekly during treatment. Toxicity was assessed not only before each vector infusion but also before beginning an additional treatment cycle. The grading of toxicity was done using the National Cancer Institute Common Terminology Criteria for Adverse Events (NCI CTCAE, 2006) version 3. Patient serum was collected for detection of vector neutralizing antibodies and antibodies to gp70. At the end of 4 weeks, peripheral blood mononuclear cells were collected to check for the presence of vector DNA integration and replication competent retrovirus (RCR), at 6 weeks, or before the start of a treatment cycle. Vector-related studies were performed as previously described (Galanis et al., 2008; Chawla et al., 2009; Chawla et al., 2019).

#### 2.5.2 Efficacy analysis

The formula for evaluation of tumor burden is given below:

Evaluation of tumor burden: Estimated tumor burden was determined for each patient using the following formula:

ETB (# cancer cells) = [Sum of Target Lesions (cm) + (No. of Non-Target Lesions + (20*)] × 10^9^ (Assumption: 1 cm = 1 × 10^9^ cancer cells).

*Note: For each instance of ascites, pleural effusion, and/or non-target lesions that are too numerous to count, 20 × 10^9^ cancer cells were considered present. (Chawla et al., 2019).

Before initiating the treatment, various imaging evaluations were conducted, which included a whole-body Fluorodeoxyglucose (FDG)-positron emission tomography (FDG PET)-computerized tomography (CT scan), electrocardiography, and chest X-ray. FDG/PET-CT scan was done for efficacy assessment at the end of 4 weeks, at the end of 6 weeks, or before starting an additional treatment cycle up to 12 weeks, and every 12 weeks thereafter. RECIST v.1.0 criteria was used to assess the tumor responses [complete response (CR); partial response (PR); stable disease (SD) or progressive disease (PD)] (Therasse et al., 2000). Tumor control rate was defined as the per cent of patients who had CR, PR or SD at any time during the DeltaRex-G treatment period. Tumor responses were also evaluated using modifications of the International PET criteria (Young et al., 1999) and the Choi criteria (Choi et al., 2007). According to the modified International PET Criteria, a CR was defined as disappearance of FDG avid uptake in target and non-target lesions with no new lesions; PR as a decrease in maximum standard uptake value of >25% from baseline with no new lesions along with no obvious progression of non-target lesions; PD as an increase in maximum standard uptake value of >25% from baseline, any new lesions, and obvious progression of non-target lesions; and SD as not meeting the criteria for CR, PR, or PD, and no symptomatic deterioration attributed to tumor progression. As per the modified Choi criteria, CR was defined as the disappearance of all evidence of disease, including any existing lesions, as well as no appearance of new lesions; PR as a decrease in size of ≥10% or a decrease in CT density (Hounsfeld units) ≥15% with no new lesions and no obvious progression of non-measurable disease; PD as an increase in tumor size of >10% and did not meet criteria for PR by CT density, any new lesions, including the formation of tumor nodules within a previously cystic tumor; and SD as not meeting the criteria for CR, PR, or PD, and no symptomatic deterioration attributed to tumor progression.

## 3 Results

### 3.1 Enrollment and demographics

Table 1 displays the characteristics of the patients. From 22 August 2007 to 24 June 2011, twenty patients participated in the study. Median age was 58.5 (range 33–77) years, 100% were women with metastatic breast carcinoma, and an ECOG score of 1. The median number of previous chemotherapy regimens received was 3 (range 1–12). Eighteen (90%) were white and 2 (10%) were Asian. Twelve (60%) patients had tumors that were HR + HER2-, 1 (5%) HR + HER2+, and 7 (35%) triple negative.

**TABLE 1:** Patient characteristics (*n* = 20).

Age (Years)	
Median	58.5
Range	33–77
Sex	
Woman	20 (100%)
Race	
White	18 (90%)
Asian	2 (10%)
Receptor	
HR + HER2-	12 (60%)
HR + HER2 +	1 (5%)
Triple negative	7 (35%)
Disease Stage	
Metastatic	20 (100%)
ECOG	1 (100%)
Number of previous chemotherapy regimens	
Median	3
Range	1–12

### 3.2 Summary of safety

No toxicity was observed that would restrict the administration of treatment at any of dose levels. Treatment related adverse events occurred in 5 patients, and almost all toxicity levels were Grade 1 or 2 except for one case. Although unrelated adverse events were reported for all patients, the incidence of these events was low (in most cases 1 or 2 occurrences per adverse event), and most were Grade 1 or 2. Three patients in the study had serious adverse events, but the Investigator did not consider them to be related to the study drug.

#### 3.2.1 Nonserious, treatment-related AEs

Table 2 shows the listing of treatment related adverse events (TRAEs). Five of the 20 patients experienced a total of 8 TRAEs. Three of the 5 patients had 1 TRAE each, 1 patient had 2 TRAEs and 1 patient had 3 TRAEs. These 8 events included chills, pruritis, pruritic rash, dry skin, and hot flushes in 1 patient each and dysgeusia in 3 patients. All study drug-related adverse events were nonserious and Grade 1 or 2 in severity, except for one event of Grade 3 pruritic rash. All of the TRAEs occurred in patients treated at Dose Level II or higher and 6 of the 8 events occurred in patients treated at Dose Level III or IV and were hypersensitivity reactions treated with diphenhydramine.

**TABLE 2:** Listing of patients with treatment-related adverse events.

System organ class	Preferred term	Number of patients	Dose level	Toxicity grade
General disorders and administration site conditions	Chills	1/7 (14%)	II	1
Skin and subcutaneous tissue disorders	Pruritus	1/6 (14%)	II	1
Skin and subcutaneous tissue disorders	Rash, pruritic	1/6 (16%)	III	3
Nervous system disorders	Dysgeusia	3/6 (50%)	III	1
Skin and subcutaneous tissue disorders	Dry skin	1/6 (16%)	III	2
General disorders and administration site conditions	Hot flush	1/6 (16%)	III	2

Notes: All drug-related AEs, were nonserious.

#### 3.2.2 Nonserious unrelated AE

The Investigator/s considered all nonserious adverse events experienced by the 20 patients to be unrelated to the study drug. The majority of unrelated events were Grade 1 or 2.

A summary of nonserious unrelated Grade 3 adverse events that were reported in more than two patients is provided in Table 3. The most frequent nonserious unrelated Grade 3 AE was vomiting (3 patients). Other Grade 3 AEs that were reported in 2 patients were anemia, nausea, AST increased, alkaline phosphatase increased, and phosphorus increased. All other Grade 3 AEs were reported in only one patient each.

**TABLE 3:** Grade 3 nonserious unrelated adverse events reported in 2 or more patients.

	Dose level			
	0-II	III	IV	Total
	*N* = 7	*N* =7	*N* =6	*N* = 20
Blood and lymphatic system disorders				
Anemia	1	1		2
Gastrointestinal disorders				
Nausea		1	1	2
Vomiting		2	1	3
Investigations				
Aspartate aminotransferase increased	1	1		2
Blood alkaline phosphatase increased		2		2
Blood phosphorus increased		2		2

Note: Numbers shown are the number of patients who experienced the indicated event at the indicated DeltaRex-G, dose level.

#### 3.2.3 Serious AEs

Three out of the twenty patients experienced serious adverse events, but they were not considered related to the study drug. These AEs comprised Grade 2 malignant pleural effusion in one patient and Grade 2 pathologic fracture in one patient. One patient had 6 SAEs: Grade 4 pulmonary embolism, Grade 4 pyrexia, Grade 4 dyspnea, Grade 4 respiratory congestion, and Grade 4 *Pseudomonas* infection and Grade 4 neutropenia with neutropenia occurring after onset of *Pseudomonas* infection. None were considered related to the study drug.

#### 3.2.4 Vector-related safety parameters

Vector-related safety parameters also indicated no adverse effects of DeltaRex-G: no patient tested positive for vector neutralizing antibodies, antibodies to gp70, replication-competent retrovirus in peripheral blood lymphocytes (PBLs) or vector integration into genomic DNA of PBLs.

### 3.3 Summary of efficacy

Table 4 shows the summary of responses to DeltaRex-G. Of the 20 enrolled and treated patients, 7 were treated at Dose Levels 0-II, 7 were treated at Dose Level III, and 6 were treated at Dose Level IV. Seventeen patients received at least one complete treatment cycle of 4 weeks and underwent follow-up PET-CT scan, thus they were considered for efficacy evaluation. By RECIST v.1.0, 13 patients had SD and 4 patients had PD. There was no clear relationship between the DeltaRex-G dosage and response, as similar numbers of patients had SD or PD at each dose level. The tumor control rate (CR + PR + SD) by RECIST was 76% (13/17 patients). By both PET and Choi modified RECIST criteria, 3 patients had PR, 11 SD and 3 PD, tumor control rate 82% indicating that PET and Choi may be indicators of early tumor response compared to the standard RECIST v1.0.

**TABLE 4:** Summary of responses in study C07-104 (Recurrent or metastatic breast cancer).

Category	Dose level^a^			
	0-II	III	IV	All
mITT Patients^b^	*N* = 6	*N* = 5	*N* = 6	*N* = 17
Median tumor burden (# cells × 10^9^)	33.8	73.9	31.0	N.D.
Median Cum. Dose (× 10^11^ cfu)	53	54	120	N.D.
Response by RECIST	5SD; 1PD	4SD; 1PD	4SD; 2PD	13SD; 4PD
Response by PET	2PR, 3SD, 1PD	1PR, 4SD	4SD, 2PD	3PR, 11SD, 3PD
Response by Choi	3PR, 3SD	4SD, 1PD	4SD, 2PD	3PR, 11SD, 3PD
Median PFS (mo) by				
RECIST	3.5	1.25	2	3
Median OS (mo)	25.6	6.75	21	
ITT population^c^	*N* = 7	*N* = 7	*N* = 6	*N* = 20
Median OS (mo)^c^	33.0	5.5	21.0	20.0
% OS				
1 year	71.4%	28.6%	83.0%	60%^d^
# Alive^e^	1/7	2/7	5/6	8/20

ITT, intent-to-treat; mITT, modified ITT; cum, cumulative; mo, month; ND, not determined; RECIST, response evaluation criteria in solid tumors; PET, positron emission tomography; Choi, modified RECIST, as described by Choi et al.PFS, progression-free survival; OS, overall survival.

^a^ Dose Level 0 = 1 × 10^11^ cfu twice per week (BIW); Dose Level I = 1 × 10^11^ cfu three times per week (TIW); Dose Level II = 2 × 10^11^ cfu TIW; Dose Level III = 3 × 10^11^ cfu TIW; Dose Level IV = 4 × 10^11^ cfu TIW.

^b^ mITT, population was defined as all patients who received at least one cycle (4 weeks) of DeltaRex-G, and had a follow-up PET CT, scan.

^c^ ITT, population was defined as all patients who received at least one infusion of DeltaRex-G.

^d^ Among patients with bone metastasis only, OS, was 100% at 2 years.

^e^ As of data cut-off date.

**FIGURE 2:**
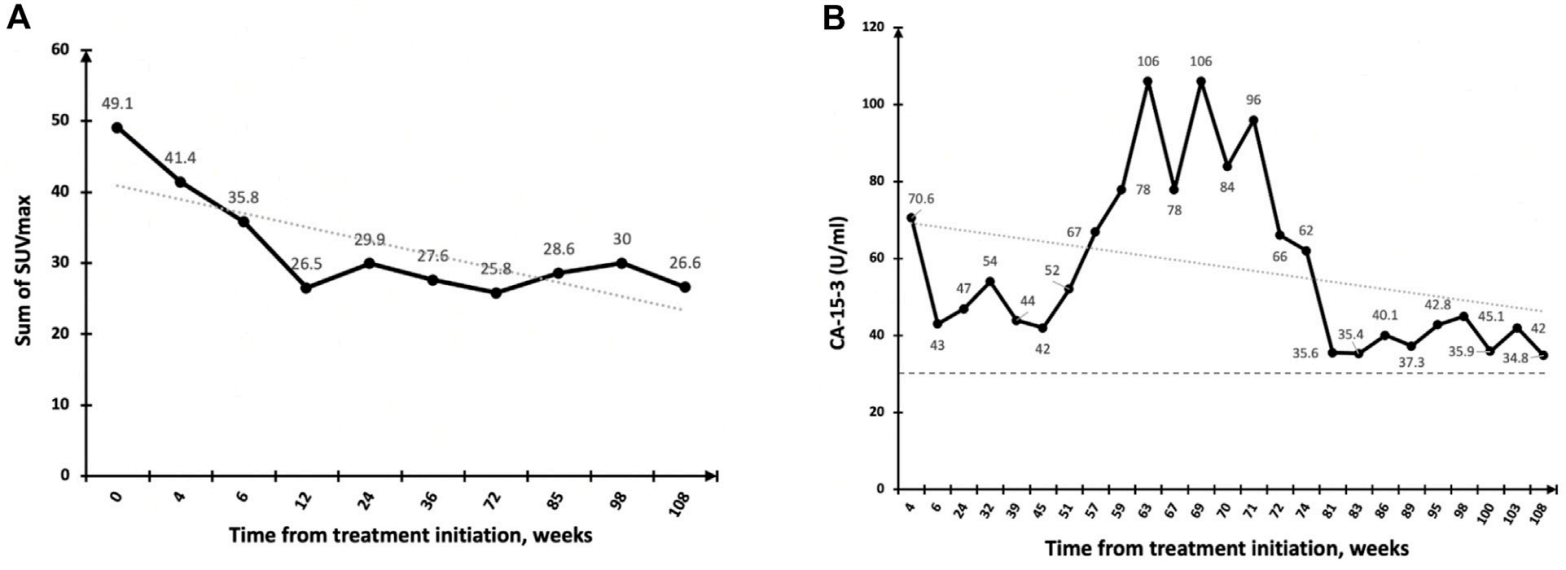
Response to DeltaRex-G in a patient with pure bone metastases. **(A)** Significant regression of bone metastases by PET-CT scan (PR) with SUVmax plotted on the vertical axis as a function of time from treatment initiation, weeks; **(B)** Fluctuating CA-15 levels plotted on the vertical axis as a function of time from treatment initiation, weeks. Normal levels are expressed as dotted lines on the 30 U/ml axis.

PFS by RECIST v1.0 ranged from 3.5 months at Dose Level 0-II, 1.25 months at Dose Level III and 2 months at Dose Level IV, thus no dose-response relationship was apparent. Patients in Dose Level III had a higher tumor burden, which may have contributed to the shorter PFS observed in this group. One of one (100%) patient with HR + HER2+ breast cancer had a PFS of 24 weeks, 5/12 (43%) patients with HR + HER2-breast cancer had a PFS ranging from 24 to 107 weeks, and one of 6 (17%) patients with TNBC had a PFS of 24 weeks. Of note, two patients (HR + HER2-) with extensive bone metastases only and no visceral involvement (one patient at Dose Level III and one at Dose Level IV) had a PFS of greater than 1 year when tested using FDG-PET (Figure 2).

OS was examined in the ITT and mITT population. OS rates in the ITT population at 1 year was 60% at all dose levels (66% in the mITT population), and 83% at Dose Level IV in the ITT and mITT populations. OS estimates at 2 years was 40%. Out of the 20 patients, eight survived for a period of 19–43 months from treatment initiation. Of those remaining alive, 1 was treated at Dose Level 0-II, 2 were treated at Dose Level III, and 5 were treated at Dose Level IV. Two patients were reported alive 3 and 12 years after treatment initiation. Of note, one additional patient (not on study) who had pure bone metastasis and received DeltaRex-G + DeltaVax (a tumor-targeted retrovector encoding a granulocyte macrophage colony stimulating factor (GM-CSF) gene) for 6 months was reported alive 12 years after DeltaRex-G treatment initiation.

## 4 Discussion

One of the exciting areas of drug development is the idea of combining targeted therapies without using chemotherapy (Rana and Sridhar, 2012; Chawla et al. 2022). The safety of multiple DeltaRex-G infusions at all four dose levels was established with no DLT in this Phase I/II study, and the MTD was not reached It is important to note that there was no treatment-related loss of hair, bone marrow suppression nor organ dysfunction at all dose levels. The serious adverse events experienced by these patients were due to disease-related complications, and were determined by the principal investigators to be unrelated to the DeltaRex-G. Additionally, no safety issues related to the vector were found. This was demonstrated by the absence of anti-vector neutralizing antibodies, antibodies to gp70, replication-competent retrovirus in PBLs, or vector integration into genomic DNA of PBLs. The data indicates that DeltaRex-G is uniquely safe when compared to FDA-approved therapies for carcinoma of breast as it has none of the dose limiting toxicities described for standard treatments (Gordon et al., 2004; Gordon et al., 2006; Gordon and Hall 2007; Gordon and Hall, 2010; Gordon et al., 2018; Chawla et al., 2009; Chawla et al., 2010; Chawla et al., 2016; Chawla et al., 2019).

**FIGURE 3:**
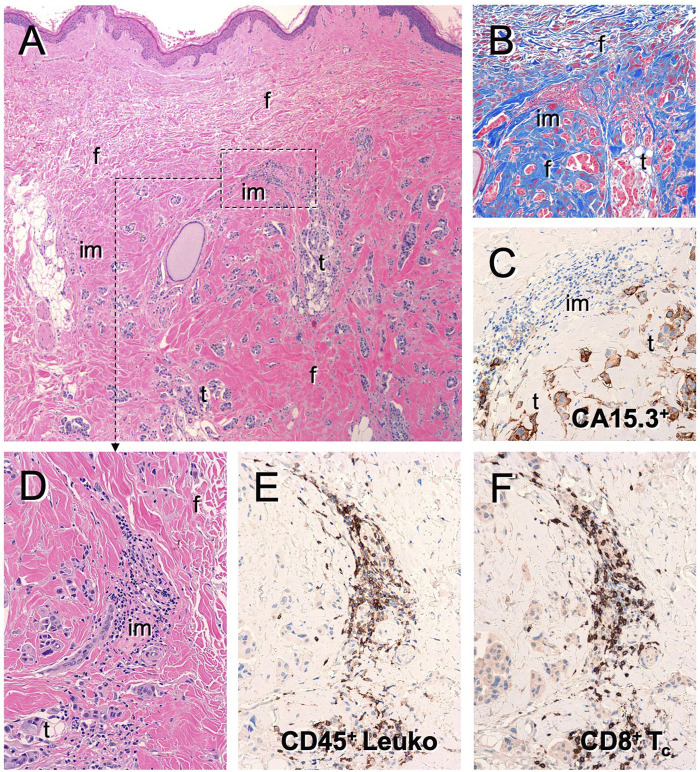
Tumor infiltrating lymphocytes in residual tumor of DeltaRex-G treated invasive breast cancer patient. Photomicrograph of resected residual tumor showing **(A)** Hematoxylin eosin stain; **(B)** Trichrome stain for collagen; **(C)** CA15-3 immunohistochemical stain for cancer cells; **(D)** Hematoxylin eosin stain showing immune cell infiltrates; **(E)** CD45^+^ cells; **(F)** CD8^+^ killer cells.

The confirmed tumor control rate of 72% by RECISTv1.0 indicates that DeltaRex-G may have anti-tumor activity in patients with metastatic breast cancer who have failed prior chemotherapy. The 1-year overall survival (OS) rate of 83% observed in patients receiving Dose Level IV is a promising finding and indicates a potential survival advantage compared to the 70% OS at 1 year in historical controls receiving first-line therapy with paclitaxel with or without lapatinib (Di Leo et al., 2008). Of note, two patients with extensive bone metastases only and no visceral involvement had the longest PFS by PET and two patients who received DeltaRex-G + DeltaVax are alive >12 years from DeltaRex-G treatment initiation. The brief treatment of the majority of patients suggest DeltaRex-G may have longer term benefits that could complement immunotherapy since histopathologic examination of a biopsied residual tumor showed presence of CD8^+^ killer cells and CD45^+^ immune cells in the tumor microenvironment (Figure 3).

This study was carried out during the period of 2007–2011. Since then, sophisticated molecular profiling for targeted therapy (Coombs et al., 2019; Bagaev et al., 2021) and immunotherapy products for metastatic breast cancer have been FDA approved at a rapid pace. However, in 2022, the American Cancer Society reported that 5-year survival for metastatic disease remained only 29% indicating that innovative therapies are urgently needed.

The clinical development of DeltaRex-G was halted in 2011 for undisclosed reasons by the acquiring entity. However, in 2018, 8 cancer survivors who were treated with DeltaRex-G with or without DeltaVax, a tumor-targeted retrovector encoding a granulocyte macrophage colony stimulating factor (GM-CSF) gene, were reported (Liu et al., 2021). This report inspired the revival of DeltaRex-G and the USFDA authorized opening of Blessed: Expanded Access for DeltaRex-G for an intermediate size population with advanced pancreatic adenocarcinoma and sarcoma, and Individual Patient Use of DeltaRex-G as adjuvant therapy for early-stage HR + HER2+ invasive breast carcinoma and triple negative breast carcinoma (Chawla et al., 2022).

### 4.1 Future perspectives

Mechanistically, DeltaRex-G blocks the cell cycle in G1 phase which causes apoptosis and necrosis of tumors, without bone marrow or innate immune suppression. Consequently, DeltaRex-G has been shown to evoke an innate immune response and white cell migration to necrotic tumors as part of the body’s clean-up process (Stendal Dy et al., 2018). The DeltaRex-G mechanism of action creates a need for caution in the interpretation of tumor responses using RECIST v.1.0 which was formulated to evaluate response to chemotherapy drugs. In our experience, DeltaRex-G causes an inflammatory clean up reaction; hence, target lesions may look larger and occult lesions may appear as new lesions on PET-CT scan in DeltaRex-G treated patients. These findings support the use of clinical judgment when evaluating tumor responses in planned Phase II/III trials as radiologic improvement could occur with extended DeltaRex-G therapy (Chawla et al., 2009; Chawla et al., 2019). Finally, the potential role of DeltaRex-G as a biochemical and/or antigen modulator for enhancing the efficacy of targeted therapies/immunotherapy is evident as DeltaRex-G does not suppress the immune system (Gordon et al., 2004; Gordon et al., 2006; Gordon and Hall et al., 2007; Gordon et al., 2018; Chawla et al., 2009; Chawla et al.2010; Chawla et al.2016; Chawla et al., 2019), and its cytocidal activity on tumor associated fibroblasts which reduces stroma production, could be harnessed to facilitate immune cell entry and/or enhance effective drug concentration into the tumor microenvironment (Stendahl Dy et al., 2018). Finally, human cyclin G1 (Gordon et al., 2018) protooncogene, a novel biomarker in development for DeltaRex-G CCNG1 inhibitor therapy, may identify patients who will respond favorably to DeltaRex-G gene therapy.

### 4.2 Limitations of the study

The phase I/II clinical trial is a non-randomized study. Therefore, more evidence would be needed from phase II or III randomized studies using DeltaRex-G (with or without immunotherapy) to reach definitive conclusions about the efficacy and safety of DeltaRex-G for metastatic carcinoma of breast.

## 5 Conclusion

Taken together, these data indicate that 1) DeltaRex-G is has a unique safety profile and has shown antitumor activity, 2) PET/Choi are more sensitive indicators of early tumor responses to DeltaRex-G, 3) The combination of DeltaRex-G with other cancer therapy/immunotherapy has the potential to act as a biochemical and/or antigen modulator, and 4) DeltaRex-G can be considered as a viable treatment option for patients who do not want to receive chemotherapy. A randomized Phase II study is warranted for further clinical development of DeltaRex-G, with or without targeted therapy or immunotherapy, for metastatic carcinoma of breast (Gordon and Hall, 2010).

## Data Availability

The raw data supporting the conclusion of this article will be made available by the authors, without undue reservation.

## References

[B1] American Cancer Society (2022). Cancer facts and figures. Atlanta, Georgia: American Cancer Society. Society.

[B2] Bagaev A. Kotlov N. Nomie K. Tsiper M. Ataullakhanov R. Fowler N. (2021). Conserved pan-cancer microenvironment subtypes predict response to immunotherapy. Cancer Cell 39, 845–865.e7. 10.1016/j.ccell.2021.04.014 34019806

[B3] Bardia A. Mayer I. A. Diamond J. R. Moroose R. L. Isakoff S. J. Starodub A. N. (2017). Efficacy and safety of anti-trop-2 antibody drug conjugate sacituzumab govitecan (IMMU-132) in heavily pretreated patients with metastatic triple-negative breast cancer. J. Clin. Oncol. 35, 2141–2148. 10.1200/JCO.2016.70.8297 28291390 PMC5559902

[B4] Baselga J. Campone M. Piccart M. Burris H. A. Rugo H. S. Sahmoud T. (2012). Everolimus in postmenopausal hormonereceptor-positive advanced breast cancer. N. Engl. J. Med. 366, 520–529. 10.1056/NEJMoa1109653 22149876 PMC5705195

[B5] Cardoso F. Paluch-Shimon S. Senkus E. Curigliano G. Aapro M. S. Andre F. (2020). 5th ESO-ESMO international consensus guidelines for advanced breast cancer (ABC 5). Ann. Oncol. 12, 1623–1649. 10.1016/j.annonc.2020.09.010 PMC7510449 32979513

[B6] Chan A. Delaloge S. Holmes F. A. Moy B. Iwata H. Harvey V. J. (2016). Neratinib after trastuzumab-based adjuvant therapy in patients with HER2-positive breast cancer (ExteNET): A multicentre, randomised, double-blind, placebo-controlled, phase 3 trial(ExteNET): A multicentre, randomised, double-blind, placebo-controlled, phase 3 trial. Lancet Oncol. 17, 367–377. 10.1016/S1470-2045(15)00551-3 26874901

[B7] Chawla S. P. Chawla N. S. Quon D. Chua-Alcala V. Blackwelder W. C. Hall F. L. (2016). An advanced phase 1/2 study using an XC-targeted gene therapy vector for chemotherapy resistant sarcoma. Sarcoma Res-Int’l 3, 1–7. id1024.

[B8] Chawla S. P. Chua V. S. Fernandez L. Saralou A. Quon D. Blackwelder W. B. (2010). Advanced Phase I/II studies of targeted gene delivery *in vivo*: Intravenous Rexin-G for gemcitabine-resistant metastatic pancreatic cancer. Mol. Ther. 18, 435–441. 10.1038/mt2009.228 19826403 PMC2839309

[B9] Chawla S. P. Bruckner H. Morse M. A. Assudani N. Hall F. L. Gordon E. M. (2019). A phase I-II study using rexin-G tumor-targeted retrovector encoding a dominant-negative cyclin G1 inhibitor for advanced pancreatic cancer. Mol. Ther. Oncol. 12, 56–67. 10.1016/j.omto.2018.12.005 PMC6348982 30705966

[B10] Chawla S. P. Chua V. S. Fernandez L. Quon D. Saralou A. (2009). Phase I/II and phase II studies of targeted gene delivery *in vivo*: Intravenous Rexin-G for chemotherapy-resistant sarcoma and osteosarcoma. Mol. Ther. 17, 1651–1657. 10.1038/mt.2009.126 19532136 PMC2835268

[B11] Chawla S. P. Wong S. Quon D. Moradkhani A. Chua V. S. Brigham D. A. (2022). Three year results of blessed: Expanded access for DeltaRex-G for an intermediate size population with advanced pancreatic cancer and sarcoma (NCT04091295) and individual patient use of DeltaRex-G for solid malignancies (IND# 19130). Front. Mol. Med. 1-10. 10.3389/fmmed.2022.1092286 PMC11285611 39086973

[B12] Choi H. Charnsangavej C. Faria S. C. Macapinlac H. A. Burgess M. A. Patel S. R. (2007). Correlation of computed tomography and positron emission tomography in patients with metastatic gastrointestinal stromal tumor treated at a single institution with imatinib mesylate: Proposal of new computed tomography response criteria. J. Clin. Oncol. 25, 1753–1759. 10.1200/JCO.2006.07.3049 17470865

[B13] Coombes R. C. Page K. Salari R. Hastings R. Armstrong A. Ahmed S. (2019). Personalized detection of circulating tumor DNA antedates breast cancer metastatic recurrence. Cancer Res. 25, 4255–4263. 10.1158/1078-0432.CCR-18-3663 30992300

[B14] Di Leo A. Gomez H. L. Aziz Z. Svirule Z. Bines J. Arbushites M. C. (2008). Phase III, double-blind, randomized study comparing lapatinib plus paclitaxel with placebo plus paclitaxel as first-line treatment for metastatic breast cancer. J. Clin. Oncol. 26, 5544–5552. 10.1200/JCO.2008.16.2578 18955454 PMC2651098

[B15] Galanis E. Carlson S. K. Foster N. R. Lowe V. Quevedo F. McWilliams R. R. (2008). Phase I trial of a pathotropic retroviral vector expressing a cytocidal cyclin G1 construct (Rexin-G) in patients with advanced pancreatic cancer. Mol. Ther. 16, 979–984. 10.1038/mt.2008.29 18388964 PMC2756987

[B16] Gordon E. M. Cornelio G. H. Lorenzo C. C. Levy J. P. Reed R. A. Liu L. (2004). First clinical experience using a “pathotropic” injectable retroviral vector (Rexin-G) as intervention for stage IV pancreatic cancer. Int’l. J. Oncol. 24, 177–185. 10.3892/ijo.24.1.177 14654955

[B17] Gordon E. M. Hall F. L. (2007). “A primer on pathotropic medicine,” in One hundred years of the FDA and the future of global Health (Shopshire United Kingdom: Brooklands New Media), 84.

[B18] Gordon E. M. Hall F. L. (2010). Rexin-G: A targeted genetic medicine for cancer. Expert Opin. Biol. Ther. 10, 819–832. 10.1517/14712598.2010.481666 20384524

[B19] Gordon E. M. Lopez F. F. Cornelio G. H. Lorenzo C. C. Levy J. P. Reed R. A. (2006). Pathotropic nanoparticles for cancer gene therapy. Rexin-G^TM^ IV: Three-year clinical experience. Int’l. J. Oncol. 29, 1053–1064. 17016635

[B20] Gordon E. M. Ravicz J. Liu S. Chawla S. P. Hall F. L. (2018). Cell cycle checkpoint control: The cyclin G1/Mdm2/p53 axis emerges as a strategic target for broad-spectrum cancer gene therapy - a review of molecular mechanisms for oncologists. Mol. Clin. Oncol. 9, 115–134. 10.3892/mco.2018.1657 30101008 PMC6083405

[B21] Hall F. L. Levy J. P. Reed R. A. Petchpud W. N. Chua V. S. Chawla S. P. (2010). Pathotropic targeting advances clinical oncology: Tumor-targeted localization of therapeutic gene delivery. Oncol. Rep. 24, 829–833. 10.3892/or.2010.829 20811660

[B22] Henry N. L. Shah P. D. Haider I. Freer P. E. Jagsi R. Sabel M. S. (2020). “abeloff’s clinical oncology,” in Chapter 88: Cancer of the breast Editors Niederhuber J. E. Armitage J. O. Doroshow J. H. Kastan M. B. Tepper J. E. (Philadelphia, Pennsylvania: Elsevier).

[B23] Jagsi R. King T. A. Lehman C. Morrow M. Harris J. R. Burstein H. J. (2019). “DeVita and rosenberg’s cancer: Principles and practice of oncology,” in Chapter 79: Malignant tumors of the breast Editors DeVita V. T. Lawrence T. S. Lawrence T. S. Rosenberg S. A. (Philadelphia, Pennsylvania: Lippincott Williams and Wilkins).

[B24] Liu S. Chawla S. P. Bruckner H. Morse M. A. Federman N. Ignacio J. G. (2021). Reporting long term survival following precision tumor-targeted gene delivery to advanced chemotherapy-resistant malignancies: An academic milestone. Clin. Oncol. 6, 1807.

[B25] Ma C. X. Sparano J. A. (2021). “Treatment approach to metastatic hormone receptor-positive,” in HER2-negative breast cancer: Endocrine therapy and targeted therapy. Editor Vora S. R. (Waltham, Massachusetts: UpToDate). Available at: https://www.uptodate.com .

[B26] Moasser M. M. Krop I. E. (2015). The evolving landscape of HER2 targeting in breast cancer. JAMA Oncol. 1, 1154–1161. 10.1001/jamaoncol.2015.2286 26204261

[B27] Mukohara T. (2015). PI3K mutations in breast cancer: Prognostic and therapeutic implications. Breast Cancer 7, 111–123. 10.2147/BCTT.S60696 26028978 PMC4440424

[B28] National Comprehensive Cancer Network (Nccn) (2021). Practice guidelines in oncology: Breast cancer. Version 6.2021. Accessed at https://www.nccn.org/professionals/physician_gls/pdf/breast.pdf (on August 13, 2021.Pembrolizumab. Reference ID: 4766009 - Accessdata. fda.gov.

[B29] Rana P. Sridhar S. S. (2012). Efficacy and tolerability of lapatinib in the management of breast cancer. Breast Cancer 6, 67–77. 10.4137/BCBCR.S6374 22438669 PMC3306225

[B30] Schmidt P. Adams S. Rugo H. S. Scheeweiss A. Barrios C. H. Iwata H. (2018). Atezolizumab and nab-paclitaxel in advanced triple-negative breast cancer. N. Engl. J. Med. 379, 2108–2121. 10.1056/NEJMoa1809615 30345906

[B31] Siegel R. L. Miller K. D. Jemal A. (2019). Cancer statistics, 2019. CA Cancer J. Clin. 69, 7–34. 10.3322/caac.21551 30620402

[B32] Stendahl Dy P. Chawla S. P. Hall F. L. Gordon E. M. (2018). Immune Cell Trafficking in the Tumor microenvironment of human cyclin G1 (CCNG1) inhibitor-treated tumors. Brit. J. Cancer Res. 1, 4. 10.31488/bjcr.117

[B33] Storer B. E. (1989). Design and analysis of phase I clinical trials. Biometrics 45, 925–937. 10.2307/2531693 2790129

[B34] The Nci Common Terminology Criteria for Adverse Events (2006). Cancer therapy evaluation program DCTD, NCI, NIH, DHHS. Available at: http://ctep.cancer.gov (Version 3(2003) 1–72.

[B35] Therasse P. Arbuck S. G. Eisenhauer E. A. Wanders J. Kaplan R. S. Rubinstein L. (2000). New guidelines to evaluate the response to treatment in solid tumors. European organization for Research and treatment of cancer, national cancer Institute of the United States, national cancer Institute of Canada. J. Natl. Cancer Inst. 92, 205–216. 10.1093/jnci/92.3.205 10655437

[B36] Xu F. Prescott M. F. Liu P. X. Chen Z. H. Liau G. Gordon E. M. (2001). Long term inhibition of neointima formation in balloon-injured rat arteries by intraluminal instillation of a matrix-targeted retroviral vector bearing a cytocidal mutant cyclin g1 construct. Intl. J. Mol. Med. 8, 19–30. 10.3892/ijmm.8.1.19 11408944

[B37] Young H. Baum R. Cremerius U. Herholz K. Hoekstra O. Lammertsma A. A. (1999). Measurement of clinical and subclinical tumour response using [18F]-fluorodeoxyglucose and positron emission tomography: Review and 1999 EORTC recommendations. European organization for Research and treatment of cancer (EORTC) PET study group. Eur. J. Cancer 35, 1773–1782. 10.1016/s0959-8049(99)00229-4 10673991

